# An Inhibitor of a Cell Adhesion Receptor Stimulates Cell Migration**

**DOI:** 10.1002/anie.201002699

**Published:** 2010-09-09

**Authors:** Shagufta H Shabbir, Jessica L Eisenberg, Milan Mrksich

**Affiliations:** Department of Chemistry, The University of Chicago, Howard Hughes Medical Institute929 East 57th Street, Chicago, IL 60637 (USA)

**Keywords:** cell adhesion, inhibitors, integrin, polyvalent interactions, self-assembled monolayers

The integrins are a family of heterodimeric receptors that play a universal role in mediating the adhesion of cells to the extracellular matrix. As such, the differential interactions of the receptors at the front and back ends of the cell are important in regulating the migration of cells.^1^ Approximately one-half of the integrins recognize the canonical RGD motif found in fibronectin, and a significant body of work has shown that model substrates presenting the RGD peptide support the adhesion and migration of cells.^2^ Inhibitors of the integrin receptor have been explored as reagents that may block cell migration and recent work has advanced a class of soluble RGD peptides as possible therapeutics that display anti-metastatic activity.^3^–^5^ But the molecular mechanisms by which these compounds block migration have not been elucidated and have recently been contradicted by work that demonstrates that the soluble antagonists can also promote the migration of tumor cells.^6^–^8^ Herein, we use a well-defined model system to assess the influence of soluble inhibitors on cell migration and we show that the integrin antagonists are able to promote the migration of cells.

The RGD-mimetic compounds cilengitide and S36578 were developed as integrin antagonists and are now in early phase clinical trials for cancer therapy.^9^, ^10^ These compounds, at micromolar concentrations, were shown to block cell migration, but recent work reported that lower concentrations of these drugs can actually enhance the growth of tumors in vivo by promoting migration and VEGF-mediated angiogenesis.^7^ Other recent work has investigated the analogy between tumor angiogenesis and wound healing^3^, ^11^ with the finding that the fibronectin-derived Pro-His-Ser-Arg-Asn (PHSRN) peptide can stimulate migration of human kerotinocytes and fibroblasts and accelerate wound healing in obese diabetic mice.^8^ Our group^12^, ^13^ and others^14^ have suggested that PHSRN antagonizes RGD binding, and therefore may accelerate migration by inhibiting the integrin-mediated adhesion. Additionally, it has been reported that addition of insulin-like growth factor binding protein (IGFBP-1) which contains the RGD sequence in the cell culture medium increases the migration of CHO cells two-fold compared to cells treated with Trp-Gly-Asp (WGD).^6^ The literature contains several additional contradictory reports as to the effect of soluble RGD-containing proteins on adhesion and migration.^5^, ^15^

Mechanistic studies of the roles of ligand–receptor interactions in cell migration are challenging because it is difficult to control which interactions operate between cell and matrix. This limitation is particularly relevant to in vivo experiments, where a broad range of signals and matrix constituents are present and not always defined.^16^ Even in the laboratory, it can be difficult to reproducibly present matrix ligands to a cell in order to obtain reproducible matrix formulations.^5^ To address these limitations, we use self-assembled monolayers that present RGD peptides against an otherwise inert background.^17^ We measure the migration profiles of large populations of individual cells and show that the addition of a soluble RGD peptide gives a dose-dependent stimulation of cell migration and we rationalize this result in terms of the properties of polyvalent adhesion (Figure 1).

**Figure 1:**
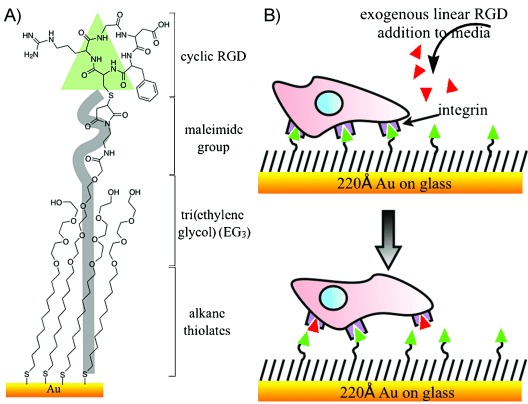
A) This work uses self-assembled monolayers (SAMs) that were prepared by immobilizing a cyclic RGD peptide to a monolayer presenting maleimide groups at a density of 1 % against a background tri(ethylene glycol) groups. B) Cells are allowed to adhere to the model surface for 2 h at which point the medium is supplemented with a linear RGD peptide. Individual cells are then tracked to determine their mean velocities for each concentration of soluble peptide.

To measure the dependence of the rate of migration of HT1080 cells on the concentration of the soluble peptide inhibitor, we allowed the cells to attach and spread on the monolayer in complete Dulbecco’s modified Eagle’s medium (DMEM) for 2 h and then supplemented the medium with the linear RGD peptide (using concentrations ranging from 0 to 500 μM) and immediately monitored the movement of cells with time-lapse microscopy for a period for 3.5 h. We prepared substrates by immobilizing cyclic RGDfC peptide to a monolayer presenting maleimide groups at a density of 1 % against a tri(ethylene glycol)-terminated background. We used a linear RGD peptide as the inhibitor in place of a cyclic peptide because the former has a lower affinity for the integrin receptor, and therefore allows a wider range of concentrations to be tested.^18^ Images of individual cells were acquired at 30 min intervals and were processed with Image-J to determine trajectories, which were then used to determine the migration rates. Figure 2 shows an example of a trajectory and illustrates that cells were able to migrate distances that correspond to several cell diameters during the experiment. We note that over 70 % of the cells remained viable during the experiment (that is, they remained attached and well spread) and we used data only for those cells that remained well spread throughout the migration assay.

**Figure 2:**
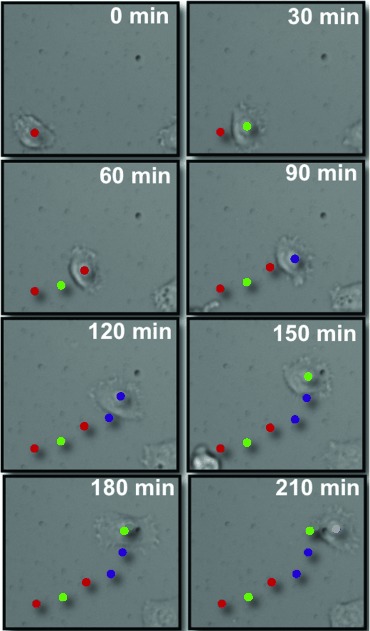
Time-lapse microscopy showing the migration of HT1080 cells on a self-assembled monolayer presenting the cyclic RGD peptide at a density of 1 % against tri(ethylene glycol) groups. Images were collected at 30 min intervals after the medium was supplemented with the linear RGD peptide (50 μM). Images were analyzed using ImageJ to create trajectories for individual cells. The images are overlaid with colored dots that represent the position of the cell at each time interval.

Table 1 summarizes the migration rates for cells treated with peptide at concentrations of 10, 25, 50, 100, 250, and 500 μM. In the absence of soluble inhibitor, cells migrated at a mean rate of 11±6.8 μm h^−1^. The rate increased to 13±5.9 μm h^−1^ in the presence of 10 μM RGD inhibitor, which was a statistically significant change as determined by Student’s *t-test* (*P*>0.05, Figure 3). The migration rate continued to increase with the concentration of inhibitor and reached a maximum of 28±11 μm h^−1^ at 100 μM. Again, a Student’s *t-test* showed that the increase in rate compared to that for cells treated with 50 μM was statistically significant (*P*>0.05). This maximum rate was 2.5-fold greater than that without inhibitor present and shows the significant influence that the inhibitor can have in promoting migration. The cell migration rates at 25 μM and 50 μM peptide were 17±7.7 μm h^−1^ and 18±7.7 μm h^−1^, respectively, which corresponded to a statistically insignificant difference (*P*<0.05). Higher concentrations of peptide (250 and 500 μM) compromised the adhesion and spreading of cells and did not yield useful migration rates. Finally, an experiment that used a scrambled RDG peptide at a concentration of 100 μM had no significant effect on the rate of migration, showing the specificity of the peptide-mediated effect. Each experiment was repeated five times.

**Figure 3:**
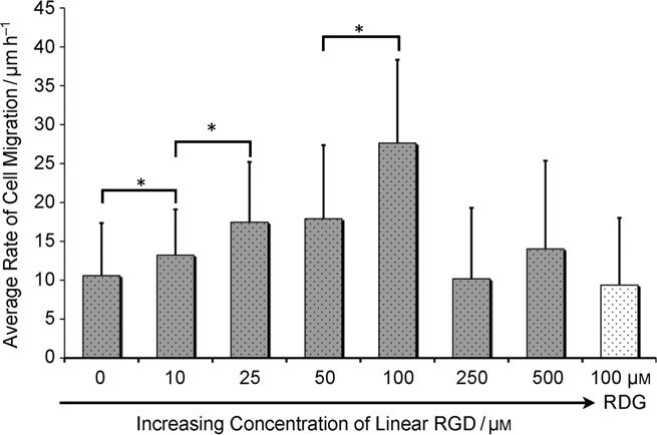
A comparison of the rates of cell migration in the presence of soluble peptide in concentrations ranging from 0 to 500 μM. Values represent the mean rate of migration with error bars determined from the standard deviation. The statistical significance of the differences in rate was determined for the following concentrations of soluble peptide: 0–10 μM (*P<0.05), 10–25 μM (*P<0.05), and 50–100 μM (*P<0.05) concentration. No statistical significance was seen between 25–50 μM (*P*>0.05). At 250 μM and 500 μM concentration of linear RGD inhibition of cell adhesion was observed.

**Table 1:** Summary of the migration experiments.^[a]^

Entry	Concentration [μM]	No. of cells	Viability [%]	Rate [μm h^−1^]	S.D. [μm h^−1^]
1	0	59	82	11	6.8
2	10	25	89	13	5.9
3	25	52	82	17	7.7
4	50	46	82	18	9.5
5	100	69	84	28	11
6	250	24	37	10	9.1
7	500	32	38	14	11
8	RDG (100 μM)	28	75	9.4	8.6

[a] The experiments were performed for concentrations of soluble peptide ranging from 0 to 500 μM and a control peptide (RDG) at 100 μM. For each experiment, the number of cells analyzed, the fraction of cells that remained viable in the culture (see text), the average migration rate, and the standard deviation (S.D.) are reported.

We suggest that the presence of a soluble ligand increases the rate of cell migration by facilitating the dynamic dissociation of the integrin receptors with the immobilized RGD ligand. Cells maintain adhesion through focal adhesions, which are clusters of integrin receptors that provide strong mechanical points of attachment to the matrix. A migrating cell must simultaneously form new associations at the front edge and turnover mature focal adhesions at the rear edge, and the slower of these two processes is expected to limit the rate of migration. Our data suggest that the addition of soluble peptide increases the rate of disengagement of focal adhesions at the rear of the cell, which would lead to a higher rate of migration if that process were the rate limiting step. This interpretation follows from the known property of polyvalent complexes to be extraordinarily stable—often mimicking irreversible association—yet which can be rapidly dissociated in the presence of a soluble ligand.^19^ The special stability arises because each of the individual interactions within the polyvalent scaffold would have to be dissociated at the same time for the complex to spontaneously dissociate, but this state is statistically improbable. Yet, the presence of a soluble ligand serves to block individual interactions from reassembling and therefore allows an “unzipping” of the polyvalent complex.

We suggest that the soluble ligands serve to disengage the focal adhesion in a similar manner. Once assembled, the focal adhesion retains a strong and nearly irreversible (in the absence of a mechanical force) association with the substrate. Dissociation of individual integrin–peptide complexes is quickly followed by reassociation. In the presence of a soluble ligand, however, the integrins are blocked by the peptide before they can reassociate with the immobilized ligand, giving a route to weaken the polyvalent adhesion, leading to its turnover. This mechanism accounts for the stimulated migration of a cell by an adhesion inhibitor and it also provides a non-intuitive strategy by which polyvalency can regulate cell behavior.

In summary, we have used well-defined substrates to address the controversial question of the effect that soluble inhibitors have on cell migration. The self-assembled monolayers present the immobilized adhesion ligand against a background that is otherwise inert and therefore allow ligand–receptor interactions to be studied in the absence of other factors that can confound the experiment. The defined and reproducible presentation of ligands allow a quantitative analysis of the migration rates of populations of cells and have lead to the clear result that a soluble inhibitor is able to stimulate migration. We believe that this and other approaches to prepare molecularly well-defined materials will allow a broader range of physical organic studies of cell function.

## Experimental Section

Preparation of self-assembled monolayer substrates: Monolayer substrates were prepared as described previously.^20^ Briefly, titanium (40 Å) and gold (220 Å) were evaporated onto glass coverslips using an electron beam evaporator (Thermionics) at a rate of 0.2–0.4 nm s^−1^ with a pressure of 1.0×10^−6^ Torr. Monolayers were formed by immersing these substrates into an ethanolic solution containing a mixture of a symmetric tri(ethylene glycol)-terminated disulfide (EG_3_) and an asymmetric disulfide with tri(ethylene glycol) and maleimide head groups for 12–16 h at room temperature . The disulfide reagents were used at a concentration of 0.5 mM with the maleimide-terminated group present at relative fractions of 1 % on the surface. All substrates were washed with ethanol and dried with a stream of nitrogen. Linear GRGDSC, GRDGS, and cyclic RGDfC were synthesized using standard Fmoc solid phase peptide synthesis protocol.^12^ Cyclic RGD was immobilized to maleimide-presenting monolayers for 2 h at 37 °C using concentrations of 100 μM, and the substrate was rinsed with 1× PBS buffer (GIBCO). The surface was characterized by SAMDI mass spectrometry. Following the peptide immobilization, the maleimide–EG_3_ peak disappears and shifts to the corresponding peptide-immobilization peak.^12^

Cell culture: HT1080 cells (ATCC) were cultured in Dulbecco’s modified Eagle medium (DMEM; GIBCO, Carlsbad, CA) supplemented with 2 mm l-glutamine (GIBCO) and 10 % fetal bovine serum (GIBCO) and 1× penicillin/streptomycin (GIBCO).

Cell migration assays: HT1080 cells were seeded at a density of 50 000 cells cm^−2^ on the 1 % cyclic RGD monolayers. The cells were allowed to adhere to the surface for 2 h at 37 °C with 5 % CO_2_, and after 2 h, a solution of linear RGD peptide (10 μM) was added. Cell migration data was recorded by computerized time-lapsed video microscopy using a CCD camera attached to an Axiovert 200 microscope from Zeiss and Openlab software (Improvisin, Lexington, MA) within a humidified chamber (37 °C, 5 % CO_2_). Images were acquired in five places on each chip through a 20× objective every 30 min for 3.5 h. The cell migration was then tracked using ImageJ (NIH) software. The experiment was repeated with 25 μM, 50 μM, 100 μM, 250 μM, and 500 μM concentration of linear RGD. As a negative control, cell migration in the presence of 100 μM concentration of linear RDG was similarly analyzed.
